# Engineered Osteochondral Scaffolds with Bioactive Cartilage Zone for Enhanced Articular Cartilage Regeneration

**DOI:** 10.1007/s10439-024-03655-1

**Published:** 2024-11-27

**Authors:** Aleksandra A. Golebiowska, Jonathon T. Intravaia, Vinayak Sathe, Sangamesh G. Kumbar, Syam P. Nukavarapu

**Affiliations:** 1 Department of Biomedical Engineering, University of Connecticut, 260 Glenbrook Road, Unit 3247, Storrs, CT 06269 USA; 2 Department of Materials Science & Engineering, University of Connecticut, Storrs, CT 06269 USA; 3 Department of Orthopaedic Surgery, University of Connecticut Health, Farmington, CT 06032 USA

**Keywords:** Decellularized cartilage, Bioactive biomaterial, Biochemical composition, Chemotactic, Zonal-structured scaffold, Tissue Engineering

## Abstract

Despite progress, osteochondral (OC) tissue engineering strategies face limitations in terms of articular cartilage layer development and its integration with the underlying bone tissue. The main objective of this study is to develop a zonal OC scaffold with native biochemical signaling in the cartilage zone to promote articular cartilage development devoid of cells and growth factors. Herein, we report the development and in vivo assessment of a novel gradient and zonal-structured scaffold for OC defect regeneration. The scaffold system is composed of a mechanically supportive 3D-printed template containing decellularized cartilage extracellular matrix (ECM) biomaterial in the cartilage zone that possesses bioactive characteristics, such as chemotactic activity and native tissue biochemical composition. OC scaffolds with a bioactive cartilage zone were implanted in vivo in a rabbit osteochondral defect model and assessed for gross morphology, matrix deposition, cellular distribution, and overall tissue regeneration. The scaffold system supported recruitment and infiltration of host cells into the cartilage zone of the graft, which led to increased ECM deposition and physiologically relevant articular cartilage tissue formation. Semi-quantitative ICRS scoring (overall score double for OC scaffold with bioactive cartilage zone compared to PLA scaffold) further confirm the bioactive scaffold enhanced articular cartilage engineering. This strategy of designing bioactive scaffolds to promote endogenous cellular infiltration can be a much simpler and effective approach for OC tissue repair and regeneration.

## Introduction

Repairing articular cartilage and osteochondral (OC) defect repair resulting from osteoarthritis poses a significant clinical problem. Osteoarthritis, a leading cause of disability affecting over 30 million people, imposes a substantial economic burden in the USA [1, 2]. This degenerative joint disease is often associated with aging, joint injury and genetic factors. Common symptoms include pain, stiffness, and reduced joint mobility, which all produce a substantial impact on quality of life. Osteoarthritis is characterized by the gradual breakdown of the articular cartilage that can advance to the underlying subchondral bone, resulting in OC defect formation [3]. The avascular nature and limited self-regeneration capabilities of dense articular cartilage makes repairing damaged OC tissue challenging, necessitating clinical intervention [4]. Common clinical treatment options for articular cartilage and OC defect repair, such as microfracture or autologous chondrocyte implantation, are often associated with donor-site morbidity, de-differentiation of cellular phenotype, late periosteal hypertrophy, and lack of integration [5, 6]. Furthermore, the limitations of the existing clinical treatments include their inability to halt the progressive nature of the disease, and limited durability of surgical intervention, often leading to fibrocartilage formation, which is mechanically inferior to native cartilage and breaks down, eventually requiring total joint replacement [7, 8].

Osteochondral tissue regeneration is inherently challenging due to the intricate composition of the OC unit, consisting of soft cartilage and hard subchondral bone tissues interconnected by a smooth interface [9, 10]. Constructing an ideal scaffold for OC tissue engineering requires meeting the distinct biological, structural, and compositional needs of each layer and their interface to facilitate the repair [11–14]. Several different scaffold configurations have been developed for OC tissue engineering (TE) with various levels of complexity, namely single phasic scaffolds or multi-phasic scaffolds, which facilitate a smoother transition between the layers of the scaffolds to mimic the distinct high level of gradient properties of the OC unit [15]. However, current TE scaffold fabrication methods face limitations in replicating the hierarchical structure of the OC interface, leading to challenges in design control and reproducibility and the ability to fabricate structures that can support the distinct phases of the OC tissue and their interface [16–18]. Despite advancements, existing challenges include addressing both chondral and osseous phases, ensuring bonding strength and mechanical integrity between/at phases, minimizing delamination, enhancing porous network interconnectivity, complexity in fabrication process and improving reproducibility while promoting cell differentiation into chondrogenic and osteogenic lineages [19]. Our previous work reported the development of different scaffold configurations for interface engineering, namely mono-phasic, bi-phasic, tri-phasic, and gradient scaffolds that were fabricated through conventional and advanced scaffold fabrication methods [20–25]. In this study, we utilized a gradient osteochondral scaffold developed via 3D printing using a single material and continuous fabrication process [20]. Polylactic acid (PLA) was selected in this study as it is a commonly used biodegradable polymer for developing 3D-printed scaffolds for tissue engineering applications. PLA serves as an example material; however, one can easily optimize the printing parameters for other known biodegradable polymers to develop the zonal structures through extrusion-based printing.

Cell-based and biological approaches have been developed to improve osteochondral repair. Cell-based TE strategies involve the use of various cell sources to promote the regeneration of both the cartilage and bone tissues [26, 27]. Common cell-based strategies rely on the use and transplantation of tissue-specific cells and progenitor cells, namely chondrocytes and osteoblasts for cartilage or bone repair, respectively, or mesenchymal stem cells (MSCs) [28–30]. However, the use of differentiated cells or stem cells comes with certain limitations, such as insufficient cellular isolation during tissue harvesting, phenotypic drift due to time consuming in vitro cellular expansion where cells undergo hypertrophy or de-differentiation and poor cell viability and survival of implanted cells [27, 31]. These often lead to suboptimal tissue regeneration or the formation of fibrous tissue, which is non-functional and mechanically inferior. Moreover, they are often faced with challenges in regard to FDA regulation, cost and scalability, off-the-shelf availability, and ethical concerns [32]. Additionally, the use of external biological factors in combination with relevant cell sources have been developed for spatial and temporal control of differentiation and phenotypic maintenance toward chondrogenic and osteogenic lineage [33, 34]. These strategies involve the use of growth factors and other signaling molecules, to promote or stimulate tissue-specific cellular responses such as osteogenesis or chondrogenesis and promote bone or cartilage regeneration, respectively [25, 35–37]. Commonly included growth factors include BMPs, TGF-$\beta$, IGF, bFGF, PDGF, and VEGF [38–41]. However, achieving and maintaining bioavailability, short half-life, limited stability, controlling release kinetics and their rapid diffusion from the delivery site, and mitigating the risk of possible adverse host reactions, including off-target effects, pose significant challenges. Additionally, addressing the high cost associated with their synthesis and accessibility and navigating regulatory challenges related to safety, efficacy, and standardization remain obstacles, limiting success in clinical translation [10, 42, 43].

Scaffold-based strategies are also developed where biomaterials are designed and engineered with appropriate structural and biomechanical properties to support and improve cellular function in order to guide subsequent tissue repair [15, 44, 45]. Due to the limitations of cellular-based strategies, research is evolving toward these acellular strategies which rely on the biomaterial to facilitate in situ tissue repair and regeneration [46]. Developing constructs that are capable of stimulating the body’s own regenerative processes at the site of injury is a promising option for in situ regeneration as it overcome many of the limitations hindering clinical translation [47, 48]. These approaches aim to create a conducive environment for tissue repair without the need for extensive *ex vivo* manipulation or cellular transplantation [49]. TE strategies often involve the use of biomaterials, growth factors, and other bioactive molecules to enhance regeneration by creating an environment that stimulates the recruitment, proliferation, and differentiation of endogenous cells for tissue repair [50, 51]. However, many of the current scaffold-based strategies are unable to recapitulate the complexity of the native ECM and lack satisfactory bio-morphological, mechanical, and bio-functional features that are critical for directing cellular behavior and leading to functional tissue repair [52]. The utilization of decellularized extracellular matrix (dECM) includes the maintenance of native tissue architecture, support for cellular adhesion, and preservation of ECM components [53–55]. Acting as a reservoir for bioactive molecules, the ECM aids in modulating cellular behavior, promoting an environment conducive to efficient tissue development and repair [56–58]. Matrices that leverage native tissue-specific biochemical and biophysical cues hold promise for improving tissue repair [59, 60]. Furthermore, ECM components have also been shown to possess chemotactic potential for stem and progenitor cells due to its ability to promote and direct cell recruitment in vitro and implant site accumulation in vivo [61–63]*.* Our recent study reported decellularized cartilage ECM induced cell recruitment and migration in 2D and 3D cell migration models using a real-time chemotaxis assay [64, 65]. Therefore, the retention of ECM components post-decellularization has the potential to stimulate endogenous cell recruitment to be used as a cell-free graft.

In this study, we report the fabrication of an engineered gradient osteochondral scaffold combined with an ECM-based hydrogel. The engineered grafts were implanted in an in vivo rabbit osteochondral defect model and assessed for endogenous cellular infiltration and tissue regeneration as a cell-free strategy for cartilage and osteochondral defect repair. We hypothesize that the use of decellularized cartilage ECM will stimulate local endogenous chondroprogenitor cellular migration and will allow for regeneration of hyaline cartilage in vivo in a rabbit osteochondral defect model, using native tissue-specific signaling molecules. Additionally, the ECM-based biomaterial in combination with a zonal-structured scaffold will provide the mechanical and structural support required for load-bearing applications, while also possessing gradient interconnected porous structure to facilitate tissue in-growth. The integrated mechanically supportive graft with the inclusion of native bioactive molecules present within the decellularized tissue may aid in the recapitulation of the native microenvironment and lead to OC regeneration in a cell-free TE approach, known as in situ tissue repair/regeneration.

## Materials and Methods

### Decellularization of Cartilage Tissue

Fresh bovine knee joints were obtained from Animal Technologies, TX (animals of age less than 30 months). Articular cartilage slices were isolated from the joints and decellularized under aseptic condition, as previously described [65]. Briefly, minced cartilage pieces were placed into a hypotonic Tris–HCl buffer solution (10-mM Tris–HCl, pH 8.0) and underwent six cycles of freezing (at − 80 °C) and thawing (at 37 °C). The tissue was then treated with 0.25% trypsin solution for 1 h at 37 °C with vigorous agitation. After trypsinization, cartilage tissue pieces were washed with a hypertonic buffer solution (1.5-M NaCl in 50 -mM Tris–HCL, pH 7.6) and treated with nuclease solution (50 U ml^−1^ DNase and 1 U ml^−1^ RNase A in 10 -mM Tris–HCL, pH 7.5) with gentle agitation at 37 °C for 4 h. To remove all the enzymes, the enzyme-treated cartilage slurry was washed with the hypotonic Tris–HCL solution for 5 h, with fresh hypotonic solution replacements every 30 min. After the nuclease treatment, tissue was treated with 1% Triton X-100 solution for 1 h. Lastly, the decellularized cartilage tissue pieces were washed with distilled water for 6 h to remove all the detergent, fresh water was replaced every 30 min. The processed tissue was then lyophilized and stored at − 20 °C until further use.

### Decellularized Cartilage Hydrogel Preparation

Decellularized cartilage ECM (dcECM) was solubilized and converted into a gel, as previously described [66]. Briefly, the lyophilized cartilage tissue pieces were ground into a powder using a micro-mill grinder (Bel-Art, H37252). The powder was then suspended at 10 mg/mL in a 0.01-M HCl solution containing 2-mg/mL pepsin for 72 h at room temperature under vigorous agitation. To remove undigested particles, the solution was then centrifuged, and the supernatant was collected. Nanoclay Laponite-XLG (BYK Additives & Instruments) powder was pre-dissolved in DI water to form a homogeneous gel. The solubilized dcECM was then combined with Laponite and a photo-curable PEG-DA (Sigma-Aldrich) along with a photoinitiator, lithium phenyl-2,4,6-trimethylbenzoylphosphinate (LAP; CELLINK). The final bioink concentration was 0.5% dcECM, 2% Laponite, 4% PEG-DA, and 0.4% LAP. The hydrogel composition was thoroughly mixed and neutralized to pH 7.4.

### Osteochondral Scaffold Fabrication

Gradient polylactic acid (PLA) scaffolds were fabricated as previously described [20]. Gradient PLA scaffolds were customized and designed with the appropriate in vivo dimensions, according to the defect created. The scaffolds were designed as cylinders of 3.5 mm diameter (using SolidWorks) with infill densities varying from 30 to 60%, as determined by the Slic3r software (with 0/90° perpendicular/alternating pattern in two consecutive layers). Three different scaffolds were designed, namely consisting of (1) 2 layers per zone, (2) 3 layers per zone, or (3) a combination of 2 or 3 layers, specifically consisting of 3 layers per zone for infill densities 30–50% and 2 layers per zone for infill densities 55–60%. An extrusion-based bioprinter, BioX (CELLINK AB), was used for the printing of the different scaffold configurations composed of polylactic acid (PLA) (CELLINK®) in a layer-by-layer fashion. PLA was printed by melting the polymer at 197 °C with 30–150 kPa pressure, printed using a speed of 3 mm s^ −1^ and a layer height of 0.160 mm. For OC graft fabrication, the previously described dcECM-based hydrogel or dcECM-based hydrogel containing PDGF-bb (50-ng PDGF-bb/scaffold) was prepared. The hydrogel was deposited directly on top of the gradient PLA scaffold, allowed to descend into the pores of the top zone of the scaffold followed by crosslinking through exposure to 405 nm irradiation for 45 s. Scaffolds were then placed into media (DMEM/F12 supplemented with 10% FBS (v/v), 1% penicillin/streptomycin (v/v)) for one day prior to the in vivo evaluation.

### Scanning Electron Microscopy (SEM) Imaging

SEM was used to image the gradient scaffolds before and after introducing the decellularized cartilage hydrogel into the cartilage zone of the OC graft. The grafts were lyophilized for 1 h prior to imaging. 3D-printed PLA template scaffolds with and without the hydrogel were sputter-coated with gold/palladium for 4 min and imaged using NanoSEM 450 (FEI). The scaffolds with the dcECM hydrogel were carefully cut down in the center to image the interior of the scaffold. This step was introduced to visualize cross-linked hydrogel layer and its presence limited to the cartilage region of the OC scaffold.

### Rabbit Osteochondral Defect Model and Surgical Procedure

**Fig. 1:**
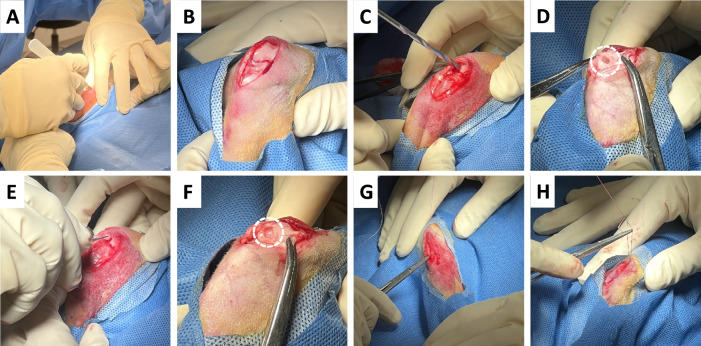
Rabbit Medial Femoral Condyle Defect Model. The main steps involved in the surgical procedure include **A** making a medial parapatellar incision, **B** exposing the medial femoral condyle, and **C** then drilling a defect in the exposed femoral condyle down to the subchondral bone of **D** approximately 3.5 mm diameter and 3 mm depth. Once the defect was made, **E**, **F** the scaffolds were implanted (where applicable) followed by **G**, **H** suturing of the muscle/soft tissue and suturing of the skin using a buried knot technique. Dotted white circles represent the location of the defect

All animal surgery protocols were approved by the Institutional Animal Care and Use Committee (IACUC Protocol AP-200301-0424) at the University of Connecticut Health. New Zealand white rabbits (30–32 weeks of age, female, weighing 3–4 kg, Envigo) were acclimated for a minimum of 7 days with ad libitum access to food and water prior to surgery. Rabbits were given a half dose of buprenorphine (0.02-0.05 mg/kg) as a pre-operative anesthesia and were anesthetized using an intramuscular injection of ketamine/xylazine/atropine and kept sedated using 2-4% isoflurane. The surgical site was shaved and then cleaned with 70% ethanol, betadine, and 70% ethanol again. For the surgical procedure, a medial parapatellar incision (2.5 cm long) was made and the medial femoral condyle was exposed (Fig. 1). A 3.5-mm defect was created by drilling down to the subchondral bone, approximately 3 mm in depth using a 3.5-mm drill bit and a handheld Aesculap® Microspeed Uni (Braun). The defects were briefly flushed with PBS to remove any debris before being filled and press fitted with one of the sample groups presented in Table 1. The muscle/soft tissue and the skin was then sutured and closed. The surgical procedure was performed on both the left and right knee of the rabbit. The groups included sham surgery, where the femoral defect is exposed but no defect was created, to serve as a positive control, as well as an empty defect group where a defect is created but no scaffold was implanted, to serve as the negative control. Six weeks after the procedure, the rabbits were sacrificed via anesthesia overdose.

**Table 1:** In vivo study groups for the rabbit medial femoral condyle defect model (n = 3)

Implant Group	
Sham surgery (positive control)	Femoral condyle is exposed but no defect is created
Empty defect (negative Control)	Defect with no implant
PLA scaffold (material Control)	Scaffolds are implanted in the femoral condyle OC defect
PLA scaffold with dcECM in the cartilage zone	
PLA scaffold with dcECM and PDGF-bb in the cartilage zone	

### Histological Analysis

Immediately after sacrifice, the gross morphology of the defect was examined and then the samples were collected. Explanted samples were fixed in 10% neutral buffered formalin for 3 days, with daily fixative changes. The samples were then decalcified using Cal-Ex (Fisher Scientific) at room temperature for 5 days, with daily decalcification solution changes. Decalcified explanted samples were then immersed in 15% sucrose solution at 4 °C overnight, followed by 30% sucrose solution overnight. Samples were then embedded in OCT embedding media and frozen at − 80 °C. Embedded samples were then cryo-sectioned at 5–10-mm-thick slices using a CM1950 cryostat (Leica) and adhered to glass slides using Norland optical adhesive 61. The slides were then cured for 5 min in the UV Stratalinker to cure the optical adhesive and stored at − 20 °C.

The sectioned slides were stained with Hematoxylin and Eosin (H&E) to visualize the general cellular and tissue morphology of the defect site. To visualize the proteoglycan content and cartilage regeneration of the defect site, the sectioned slides were stained with Safranin O/Fast Green/Weigert’s Hematoxylin or with Toluidine Blue. The stained slides were then cover slipped and imaged using brightfield microscopy (IX83, Olympus). Three independent evaluators blindly scored the surface smoothness, matrix, and cellular distribution of the defect area using the International Cartilage Repair Society (ICRS) scoring system (Table 2), using a total of 18 rectangular regions of interest per group [67–70]. Group scores were calculated as the average total score determined by the evaluators.

**Table 2:** International Cartilage Repair Society (ICRS) scoring system used for the evaluation of cartilage repair after 6-week implantation in a rabbit femoral condyle defect model

Feature	Score
I. Surface	
Smooth/continuous	3
Discontinuities/irregularities	0
II. Matrix	
Hyaline	3
Mixture: Hyaline/fibrocartilage	2
Fibrocartilage	1
Fibrous tissue	0
III. Cell distribution	
Columnar	3
Mixed/columnar clusters	2
Clusters	1
Individual cells/disorganized	0
IV. Overall score	9

### Immunofluorescent Staining Analysis

Immunofluorescent staining for collagen type I, II, or X was also performed. Briefly, the slides were treated with Pronase (1 mg/mL in PBS) for 30 min at 37 °C, followed by Hyaluronidase (5 mg/mL in PBS) for another 30 min at 37 °C. Samples were then treated with 10% normal goat serum for 1 h at room temperature. Afterward, the samples were treated with the primary antibodies in a humidified chamber at 4 °C overnight. Primary antibodies used included mouse monoclonal antibodies, anti-Collagen I antibody (ab6308, 1:400 dilution, Abcam), anti-Collagen II antibody (ab185430, 1:100 dilution), and anti-Collagen × antibody (ab49945, 1:500 dilution, Abcam) to determine the specific type of cartilage development. After overnight incubation, the slides were treated with Goat Anti-Mouse IgG H&L (Alexa Fluor® 647) (ab150115, 1:500 dilution, Abcam) for 1 h at room temperature. The stained slides were then cover slipped and imaged using a fluorescent microscope (IX83, Olympus) using 650/665-nm excitation/emission wavelengths.

### Statistical Analysis

Quantitative data was reported as mean ± standard deviation. A one-way analysis of variance was used where appropriate, with Tukey’s *post hoc* test at a significance level of *p* < 0.05 in all the statistical tests performed.

## Results

**Fig. 2:**
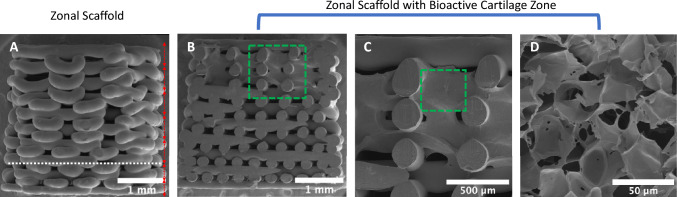
3D-printed and zonal OC graft Fabrication. **A** SEM images of the resulting gradient osteochondral scaffold for in vivo implantation consisting of a combination of zonal layers (3 layers per zone for the top five zones and 2 layers per zone for the bottom two zones, as shown) (dotted white line shows the separation between the 3 and 2 layer zones, dotted red arrows identify individual zones). The fabricated scaffold is 3.5 mm by 3 mm (diameter by height). **B–D** SEM images of the resulting OC graft following the infusion of the hydrogel within the gradient scaffold at three different magnifications, showing the presence of the hydrogel, which remains confided to the top cartilage zone (1 mm). Dotted green lines represent the areas of higher magnification

We previously report the fabrication of a series of multi-zonal and gradient structures using 3D printing for osteochondral tissue engineering [20]. The OC scaffolds were developed as bi-phasic, tri-phasic, and gradient scaffolds to support the zonal and hierarchical structure of the OC tissue. In this study, the gradient scaffolds were selected for the current in vivo study as it possesses a graded structure which allows for the smooth transition from cartilage phase to bone phase, rather than an abrupt transition, which happens with the bi-phasic or tri-phasic OC grafts. Furthermore, these scaffolds feature variations of pore sizes and porosity, increasing from the bottom to the top of the scaffolds of equal heights per zone, designed as the articular layer (top zone) and the underlying subchondral bone layer (bottom zone). However, in order to utilize the previously designed gradient scaffolds for the in vivo study, the scaffolds’ dimensions were modified according to the size of the defect that was created. In this study, gradient scaffolds were designed and fabricated as cylinders of 3.5 mm in diameter and to contain either 2 layers per zone or 3 layers per zone. These structures led to the development of scaffolds with a height of 2.24 and 3.36 mm for the 2 layers and 3 layers per zone, respectively (not shown here). As both scaffolds did not meet our height requirements for our in vivo study, a combination of 2 or 3 layers per zone was adopted. Scaffolds were designed to contain 3 layers per zone for the top 5 zones of the scaffold and 2 layers for the bottom 2 zones, resulting in scaffolds developments with the dimensions of 3.5 mm in diameter and 3 mm in height (as shown in Fig. 2A). SEM imaging showed these final scaffolds maintained their ability to establish graded zonal development. Furthermore, these scaffolds were designed to facilitate or allow for the combination of a secondary biomaterial within desired zones of the PLA template. Herein, the ECM-based hydrogel was combined within our gradient scaffolds for the formation of OC scaffolds with dcECM in the cartilage zone (Fig. 2B–D). SEM imaging showed the presence and complete infusion of the hydrogel within the top zone of the scaffolds, which remained confided to the top zone (1 mm). The incorporation of the ECM-based hydrogel led to the development of OC grafts with bioactive cartilage zone.

**Fig. 3:**
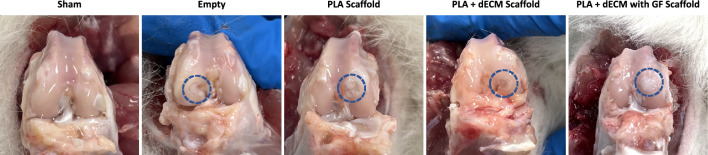
OC explant gross morphological examination. Representative gross morphology images of rabbit femoral condyles after 6 weeks for sham surgery, empty defect, PLA scaffold, PLA + dECM, and PLA + dECM with GF scaffold groups. The images of the defect sites reveal no evidence of inflammation, infection or necrotic tissue and showed defect filling (partial to near complete) for all groups. Blue-dotted circles represent the defect sites

To assess the cartilage regeneration potential of the developed OC grafts, a 6-week pilot rabbit femoral condyle defect in vivo study was performed. The gross morphological appearance of the defect areas revealed that all sample groups did not show any evidence of inflammatory reaction, infection, or necrotic tissue responses (Fig. 3). Moreover, the defect filling observed after 6 weeks for all groups varied. The defect sites were visibly evident with neotissue distinctive from the surrounding native cartilage. The empty defect and the PLA scaffold groups revealed incomplete defect filling with noticeable depressions as well as presence of fibrous-like tissue formation and lack of integration. Whereas the PLA scaffold with decellularized cartilage ECM group showed uniform and partial defect filling with neotissue that showed a smooth surface and coloration similar to the surrounding tissues with only partial integration. It is noteworthy that the PLA + dcECM with GF scaffold group showed near-complete and semi-uniform defect filling compared to the control groups, therefore demonstrated better integration with the surrounding native cartilage.

**Fig. 4:**
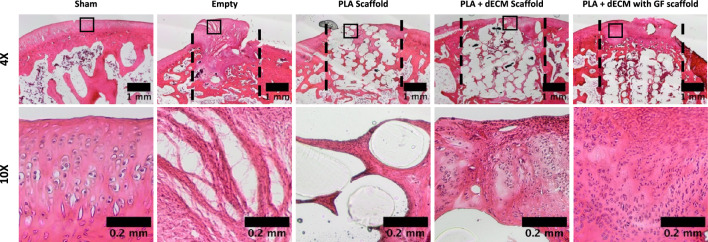
Histological evaluation of the tissue stained with H&E after six weeks. Representative H&E staining of the rabbit osteochondral defect sites used to visualize cellular and tissue morphology for sham surgery, empty defect, PLA scaffold, PLA + dECM scaffold, and PLA + dECM with GF scaffold groups. Images show unorganized tissue for the empty defect group and minimal tissue formation and cellularization for the PLA scaffold group. The PLA + dECM without and with GF scaffold groups show formation of cell rich, mostly continuous upper layer. Black-dotted lines represent the defect sites

Histological analysis using H&E staining revealed differences in cellular and tissue morphology of the defect filling 6-week post-implantation (Fig. 4). For the empty defect, tissue filling was mostly with fibrous tissue, which is characterized by irregular, thin and unorganized tissue formation with elongated and spindle-shaped cells located through the defect area. The material control, PLA scaffold group, showed minimal cellular infiltration with similar elongated cells and very little neotissue formation that was limited to the peripheries of the defect. Both the PLA + dECM scaffold and the PLA + dECM with GF scaffold implantations showed complete cellularization throughout and into the interior of the upper zone of the scaffolds. This observation also revealed the formation of a cell-rich and seemingly organized neotissue formation that was more similar to the native tissue (sham), compared to the empty defect and PLA scaffold group. H&E staining of the PLA + dECM scaffold showed the development of a semi-continuous layer that was partially integrated with the surrounding native tissue. Whereas, the PLA + dECM with GF scaffold group exhibited the development of a mostly continuous layer that was better integrated with the surrounding tissue.

**Fig. 5:**
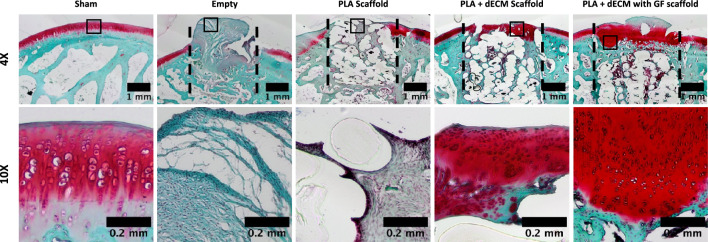
Histological evaluation of the tissue with Safranin O after six weeks. Representative Safranin O staining of the rabbit osteochondral defect sites used to visualize proteoglycan content for sham, empty defect, PLA scaffold, PLA + dECM scaffold, and PLA + dECM with GF scaffold groups. Images show little to no Safranin O staining for the empty defect and PLA scaffold groups. PLA + dECM groups without and with GF showed formation of articular cartilage matrix regeneration with differences in cellular arrangement/organization and integration with the host tissue. Black-dotted lines represent the defect sites

**Fig. 6:**
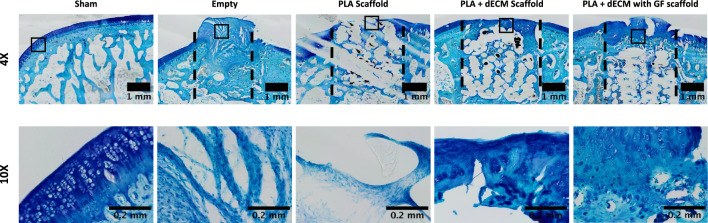
Histological evaluation of the tissue with Toluidine Blue after six weeks. Representative Toluidine Blue staining of the rabbit osteochondral defect sites used to visualize the cartilaginous tissue regeneration for sham, empty defect, PLA scaffold, PLA + dECM scaffold without and with GF scaffold groups. Images confirm the histological staining with Safranin O and show little to no Toluidine Blue staining for the empty defect and PLA scaffold groups. PLA + dECM scaffold groups without and with GF showed formation of cartilaginous tissue regeneration with differences in the formation of the upper layer. Black-dotted lines represent the defect sites

Next, Safranin O/Fast Green and Toluidine Blue were utilized for the detection and visualization of developed articular cartilage, and these stains specifically bind to the proteoglycan content in the repair tissue as shown in Figs. 4 and 5. For the empty defect group, the Safranin O and Toluidine Blue staining showed the lack of cartilaginous matrix as evidenced by the absence of proteoglycan staining. The PLA scaffold group showed minimal presence of Safranin O and Toluidine Blue staining, which was mostly isolated or located in the peripheries of the defect site and thus, the lack of cartilage regeneration. For both the PLA + dECM scaffold and the PLA + dECM with GF scaffold groups, there were higher levels of both Safranin O and Toluidine Blue staining, compared to the empty defect and PLA scaffold groups. The PLA + dECM scaffold group exhibited the development of a semi-intact cartilaginous matrix, with some presence of disruptions within the cartilaginous phase of the scaffolds and minimal connectivity with the surrounding tissue. However, the PLA + dECM scaffold group also exhibited the semi-moderate presence of cellular clusters and disorganized distribution of cells within the upper portion of the defect area. In contrast, the PLA + dECM with GF scaffold group showed minimal to lack of presence of cellular clusters. Additionally, this scaffold group showed the presence of rounded chondrocyte-like cells encased in pericellular lacunae that were arranged vertically in columns within the deep zone, which is more similar to that seen in the native tissue (sham) group. Toluidine Blue staining of the PLA + dECM with GF scaffold also revealed the possible presence of tidemark formation. Furthermore, the PLA + dECM with GF scaffold group demonstrated the development of a cartilaginous matrix formation that was continuous and better integrated with the surrounding native tissue in the upper portion of the cartilaginous phase of the scaffolds (Fig. 6).

**Fig. 7:**
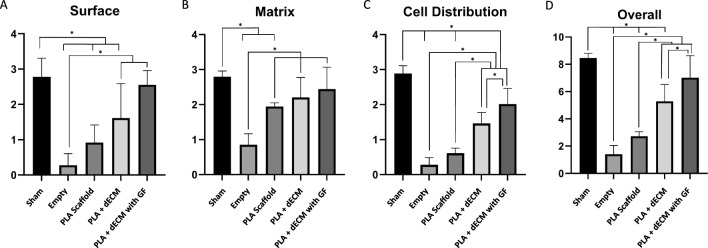
Semi-quantitative scoring assessment of the cartilage tissue repair quality after six weeks. Histological staining scoring for the sham, empty defect, template scaffold, PLA + dECM scaffold, and PLA + dECM with GF scaffold groups using the ICRS scoring system for **A** Surface, **B** Matrix, **C** Cellular distribution, and **D** Overall assessment

Histological assessment using the ICRS scoring system for cartilage tissue repair showed differences in the repair quality of the tissue according to the features evaluated (Fig. 7) [67–70]. For surface evaluation of the defect repair after 6 weeks showed that the PLA + dECM with GF scaffold group had the highest scores, 2.56 ± 0.40, when compared to the sham group, 2.78 ± 0.17, followed by PLA + dECM scaffold group, 1.61 ± 0.97, the template group, 0.92 ± 0.5, and the empty defect group, 0.28 ± 0.33. A similar trend was observed for the matrix and cellular distribution evaluation of the cartilage repair quality. For matrix evaluation, although not statistically significant, the PLA + dECM with GF scaffold group showed the highest scores of 2.44 ± 0.62, second only to the sham group, 2.80 ± 0.16, followed by the PLA + dECM scaffold group, 2.20 ± 0.98, the template group, 1.19 ± 0.11, and lastly the empty defect group which received scores of 0.85 ± 0.33. For cellular distribution, the PLA + dECM with GF scaffold group showed significantly higher values compared to the PLA + dECM scaffold group, template scaffold group, and empty defect group. Lastly, a similar trend was also demonstrated for the overall scoring assessment of the cartilage repair quality after 6 weeks where the PLA + dECM with GF scaffold group demonstrated significantly higher scores compared to the PLA + dECM scaffold group, template scaffold group, and the empty defect group.

**Fig. 8:**
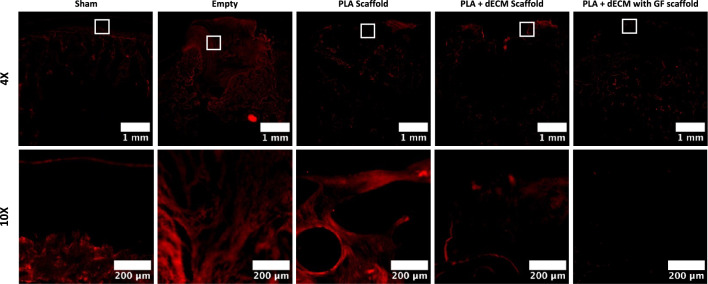
Immunofluorescent staining of collagen type I after six weeks. Representative collagen type I staining images of the rabbit osteochondral defect sites for sham, empty defect, PLA scaffold, PLA + dECM scaffold, and PLA + dECM with GF scaffold groups. Images show that the presence of collagen type I was mainly seen in the empty defect group and the PLA scaffold groups. The PLA + dECM scaffold group showed minimal presence of collagen type I in the cartilage layer, whereas no collagen type I presence was detected in the PLA + dECM with GF scaffold group

For more accurate determination of the type of cartilage tissue that was regenerated in the defects, immunofluorescent staining for collagen type I, collagen type II and collagen type X was performed to represent presence of fibrocartilage, hyaline-like cartilage, and hypertrophic cartilage, respectively. Immunofluorescent staining for collagen type I for the empty defect group revealed the abundant positive staining throughout the defect site and the presence of fibrous, unorganized tissue formation (Fig. 8). Likewise, PLA scaffold group also revealed the presence of collagen type I throughout the defect site/scaffold and especially near the peripheries of the defect. The PLA + dECM scaffold group showed presence of collagen type I in isolated areas of the defect site, mostly within the peripheries of the defect and within the deep zone of the cartilaginous phase extending to the deeper zones of the scaffold. However, the PLA + dECM with GF scaffold group exhibited minimal levels of collagen type I formation in the upper regions of the defect, in comparison to the other groups.

**Fig. 9:**
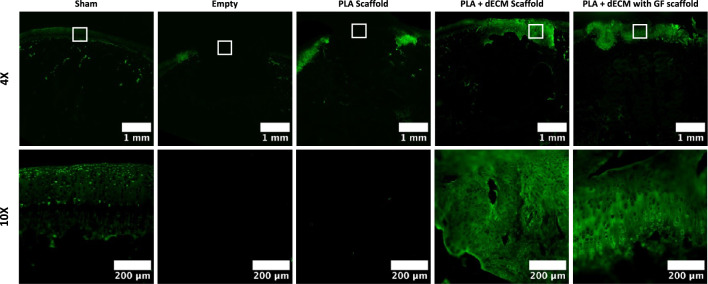
Immunofluorescent staining of collagen type II after six weeks. Representative collagen type II staining of the rabbit osteochondral defect sites for sham, empty defect, PLA scaffold, PLA + dECM scaffold, and PLA + dECM with GF scaffold groups. Images showed little to no presence of collagen type II in the empty defect and PLA scaffold group. An abundance of collagen type II staining was detected in the PLA + dECM scaffold and PLA + dECM with GF scaffold groups

Immunofluorescent staining for collagen type II, for both the empty defect and the PLA scaffold group, remained largely negative and did not show any evidence of collagen II staining (Fig. 9). Meanwhile, the immunofluorescent staining revealed significantly increased deposition of collagen type II in both the PLA + dECM scaffold and the PLA + dECM with GF scaffold groups, compared to the empty defect and the PLA scaffold groups. Both groups (PLA + dECM without and with GF scaffolds) revealed the abundance of collagen type II expression and staining within the cartilaginous phase corresponding to the higher levels of proteoglycan staining, seen previously in Fig. 5. Differences in the cellular organization were also revealed in these groups, where the PLA + dECM scaffold group exhibited lack of cellular organization throughout the upper cartilage region. Conversely, the PLA + dECM with GF scaffold group revealed a more organized cellular distribution and the arrangement of cellular columns within the deep zone of the upper cartilaginous zone. Lastly, immunofluorescent staining for collagen type X showed minimal to no expression of collagen X in any of the scaffold groups or the empty defect group (supplemental data).

## Discussion

Osteochondral scaffold development utilizes a range of different biomaterials from natural and synthetic-based polymers, many fabrication methods, and various architecture designs, each of which possess their own unique challenges and opportunities [71–75]. Fabrication method and biomaterial selection must be carefully considered to mimic the hierarchical and zonal characteristics of the native OC unit to provide a suitable microenvironment for cells to stimulate tissue regeneration while providing compatible mechanics and bioactivity properties [12, 76–78]. Furthermore, fabrication methods need to be devised such that the scaffold shape and dimensions can precisely match the osteochondral defect and have the ability to be tailored.

In this study, we investigated the use of decellularized cartilage ECM-based hydrogel in combination with a mechanically supportive gradient scaffold for osteochondral engineering. The mechanically supportive and continuous gradient scaffold was utilized in this work to withstand physiological forces in load-bearing applications, such as in OC defect repair, and to mimic the zonal structure of the native OC tissue more closely through its continuous spatial transition in scaffold properties. Whereas, the bioactive ECM-hydrogel is designed to enhance cellular responses, stimulate and maintain tissue repair processes, and aid in cellular recruitment, infiltration, and differentiation. Fabrication of these scaffolds via 3D printing allowed for customization of these scaffolds with the appropriate in vivo dimensions for our rabbit osteochondral study, which is considerably smaller compared to other models. Furthermore, the internal pore architecture of the gradient scaffold structure allowed for incorporation of our bioactive hydrogel, resulting in the hierarchical stratification and the zonal spatial distribution of biological and chemical cues confined to the cartilage zone of the OC scaffolds.

To assess the cartilage regeneration potential of the OC scaffolds developed in this study in an in vivo environment, a pilot rabbit osteochondral defect model was utilized. Histological assessment of the PLA scaffold group showed little to no cellularization due to the absence of the hydrogel and lack of a microenvironment to support cellular adhesion, whereas the empty defect demonstrated spontaneous, non-targeted cellular infiltration, which led to fibrous-like tissue formation. However, osteochondral defects implanted with the PLA + dECM without and with GF scaffold groups showed complete cellularization throughout the cartilaginous zone of the OC scaffolds. PLA + dECM without and with GF scaffold groups induced the formation of the cell-rich layer, which is associated with seemingly organized neotissue formation, unlike the empty defect and PLA scaffold group. Generation of the highly cellularized region of these scaffolds is likely due to the release of embedded chemotactic and bioactive cues through the degradation of the biomaterial from within the upper cartilaginous region of the OC scaffold. These results are in line with previous studies which have shown the potential of decellularized ECM to stimulate the migration of endogenous cells [65]. These results indicate that both PLA + dECM without and with GF scaffold groups are capable of recruiting the surrounding endogenous cells and stimulating their migration into the defect site after they are implanted in vivo.

The potential for recruited endogenous cells to restore cartilage defects requires the establishment of a chondrogenic microenvironment, which is essential for achieving successful outcomes. The PLA + dECM without GF scaffold groups showed evidence of cartilage regeneration with minimal integration seen through Safranin O and Toluidine Blue staining for proteoglycan deposition and collagen type II accumulation within the upper cartilaginous zone of the scaffolds. These findings suggest that the ECM-based hydrogel as part of the OC scaffold not only facilitated the recruitment and infiltration of endogenous stem cells within the scaffold but also provided a favorable chondrogenic microenvironment for tissue repair. However, the PLA + dECM without GF scaffold groups also showed presence of collagen type I staining, along with cell clusters and disorganized cellular distribution. The presence of the collagen type I may indicate the beginning of fibrocartilage formation, inferior quality of repair tissue [79, 80]. Meanwhile, the presence of cell clusters, composed of at least three chondrocytes, may indicate early changes of a subpopulation of cartilage cells to their phenotype, leading the cells to undergo activation and proliferation [81–83]. There are some reports in the literature that cluster formation may be associated with phenotypic drift and hypertrophy within the cartilage, while other studies report the development of degenerative changes or OA [84, 85]. Although PLA + dECM without GF scaffold groups stimulated both cellular recruitment and cartilage-like tissue formation, they may have also led to the recruitment of a mixed differentiation phenotype and may require a longer timepoint to evaluate the progression of the defect repair.

The defect repair for the PLA + dECM with GF scaffold group also showed evidence of cartilage regeneration. This group demonstrated that scaffolds loaded with GF could induce migration and chondrogenesis of recruited cells as seen through increased proteoglycan and collagen type II deposition, without the presence of fibrocartilage. Moreover, these scaffolds exhibited more mature cartilage formation with typical lacunae structure, cellular organization, and early indications of tidemark formation. It is noteworthy that there is no presence of cell clusters seen through Safranin O and Toluidine Blue staining. Additionally, the PLA + dECM with GF scaffold group showed the highest scores when evaluated for all features (surface, matrix, cellular distribution, and overall) compared to the PLA + dECM scaffold group, template scaffold group, and empty defect group. Furthermore, the PLA + dECM with GF scaffold group showed the most similar scores compared to the sham group. It is evident from the ICRS data that the cartilage repair quality of the PLA + dECM with GF scaffold group outperformed the other three groups, with similarity to the native cartilage tissue. The ECM possesses the inherent structural, biochemical, and biomechanical cues of the native tissue; however, the decellularization process of tissues and organs often results in the decreased retention of these ECM components, including signaling molecules, such as growth factors [86–89]. In order to compensate for the loss of these ECM components during processing, the scaffolds were developed with drug-loading capabilities through Laponite. We previously showed that these ECM-based hydrogels facilitated the binding and sustained release of loaded GF. PDGF-bb, in particular, was utilized for these studies as the GF has been reported to promote cartilage tissue regeneration as it is a potent mitogenic and chemotactic factor for various cells, including MSCs and chondrocytes [65, 90–93]. Moreover, the incorporation of PDGF-bb has also been shown to improve in vivo cartilage repair [94–96]. Studies have demonstrated that collagen sponge scaffolds impregnated with PDGF or hydroxyapatite/silk fibroin scaffolds that supported the continuous release of PDGF-bb led to improved repair of full-thickness OC defects [97, 98]. The system developed as part of this work allowed for the enrichment of the ECM-based hydrogel or cartilaginous phase of the OC gradient scaffolds. Moreover, the enriched system likely facilitated the stable loading, and localized controlled release from the scaffold for targeted recruitment and in situ tissue regeneration of the defect area. Indicating that the presence of both the ECM-based hydrogel and the GF, PDGF-bb, led to the cellularization of the top zone of the OC scaffolds with the appropriate and homogeneous type of endogenous chondroprogenitor cells and their ultimate differentiation and organization, that represents typical chondrocyte features and enhanced the repair ability of the cartilage defects.

## Conclusion

In conclusion, we developed a zonal-structured scaffold with gradient porosity and mechanical stability using 3D printing. The template scaffold is combined with decellularized cartilage extracellular matrix-based biomaterial to form OC grafts with a bioactive cartilage zone. The fabrication process is optimized to develop OC cylindrical structures in varied sizes while preserving gradient porosity and zonal-structured configuration. The OC grafts, with or without growth factors in the cartilage zone, implanted in rabbit osteochondral defects facilitated cellularization of the top cartilaginous zone through endogenous cellular recruitment, resulting in cartilage regeneration. However, the OC scaffold with the inclusion of PDGF-bb demonstrated repaired cartilage defects that were fully covered with normal hyaline, articular cartilage-like tissue formation with mature chondrocytes as early as 6 weeks. Qualitative and quantitative histological staining analysis indicated healthy cartilage formation for the OC scaffolds with the bioactive cartilage zone. The OC scaffolds developed in this study could function independently as a regenerative strategy, showing comparability or superiority to other methods involving the delivery of cells and bioactive factors as an *in situ* tissue engineering approach for cartilage and osteochondral defect repair. However, ongoing studies are evaluating OC scaffolds with multiple bioactive zones corresponding to bone, cartilage, and bone-cartilage interface with the aim of characterizing regenerated OC tissue qualitatively and quantitatively for tissue quality, structure, and function.
